# Predictive factors of ^18^F-choline PET/CT positivity in patients with prostate cancer recurrence after radiation therapy: is the impact of PSA nadir underestimated?

**DOI:** 10.1186/s13550-016-0237-0

**Published:** 2016-11-21

**Authors:** Alison C. Johnson, Audrey Emmanuelle Dugué, Marlon Silva, Laura Moise, Xavier Tillou, Florence Joly, Nicolas Aide

**Affiliations:** 1 Department of Oncology, François Baclesse Cancer Center, Caen, France; 2 Department of Radiation Therapy, François Baclesse Cancer Center, Caen, France; 3 Department of Urology, University Hospital of Caen, Caen, France; 4 Department of Nuclear Medicine, University Hospital of Caen, Caen, France; 5 Department of Nuclear Medicine, François Baclesse Cancer Center, Caen, France

**Keywords:** Biochemical recurrence, Radiation therapy, ^18^F-choline PET, Prostate cancer, PSA nadir

## Abstract

**Background:**

The objective of this study is to explore the impact of PSA nadirs on detection rates of prostate cancer (PCa) recurrence with ^18^F-choline (CH) PET/CT after external beam radiation therapy (EBRT).

**Methods:**

In this retrospective study, data were collected from 54 patients with suspicion of PCa biochemical recurrence after EBRT (28 patients treated initially with EBRT and 26 as salvage therapy in the absence of PSA decrease after initial treatment), who underwent ^18^F-CH PET/CT between 2010 and 2015. PSA nadir and trigger PSA were collected from patient files. Relative PSA was calculated by subtracting the nadir from the trigger PSA.

**Results:**

Median PSA nadir was 0.31 (0.01–13.31) ng/mL, trigger PSA was 7.85 (0.47–111.60) ng/mL, and relative PSA was 6.05 (0.24–104.59) ng/mL. Overall, 40 (74%) PET/CT scans were positive: recurrence was local and/or regional in 29 patients, distant in 15 and combined both in four, with no association between PSA values and sites of recurrence.

In univariate analysis, trigger (*p* = 0.015) and relative (*p* = 0.0005) PSA values and PSA velocity (*p* = 0.01) were significantly linked to positive PET/CT, but PSA nadir was not. In subgroup analysis, these significant differences were only found in the salvage EBRT group. Akaike Information Criterion multivariate model comparison found that relative PSA was a better predictor of positive PET/CT than trigger PSA (PSAt).

^18^F-CH PET/CT detection rates increased with trigger and relative PSA: 0% (0/4 patients), 71% (5/7 patients), and 81% (35/43 patients) for PSAt <2 ng/mL, 2≤ PSAt ≤4 ng/mL, and PSAt >4 ng/mL, respectively, and 14% (1/7 patients), 50% (5/10 patients), and 92% (34/37 patients) when relative PSA was taken into account instead of trigger PSA, with seven (13%) patients changing subgroups.

**Conclusions:**

We found a high overall detection rate and an increase in detection rates proportional to trigger and relative PSAs. Although relative PSA, taking into account PSA nadir, was a better predictive factor of PET/CT positivity in univariate analysis, this was most noticeable for high PSAs. For low PSAs, trigger PSA remains most relevant. Larger series with intermediate PSA values need to be studied to fully apprehend nadir impact.

## Background

Prostate cancer (PCa) is the most common cancer in elderly men in developed countries and the fifth leading cause of cancer-related death worldwide [1]. Known significant risk factors include age, heredity, and ethnicity. Risk groups defined by baseline prostate-specific antigen (PSA), TNM staging, and Gleason score help guide treatment [2]. Treatment options also depend on age, life expectancy, and quality of life. Patients with clinically localized disease can be treated with radical prostatectomy (RP) or external beam radiation therapy (EBRT) alone or with androgen deprivation therapy (ADT). Low-dose rate brachytherapy is an option for certain low-risk PCa patients [3].

Pre-treatment nomograms are available to determine the risk of biochemical recurrence after RP [4] and EBRT [5]. At 10 years of follow-up, approximately 35% of men treated with RP and 50% of men treated with EBRT will develop biochemical recurrence [6], defined as two consecutive increasing PSA values >0.2 ng/mL after RP and >2 ng/mL above the nadir after EBRT [7].

Salvage treatment is adapted to recurrence confirmation and staging (local, regional, or distant). Rising PSA is the main tool for PCa follow-up, but cannot predict the probability of systemic disease. Conventional imaging modalities such as bone scintigraphy, computed tomography (CT), and magnetic resonance imaging have relatively low accuracy and diagnostic yield in asymptomatic patients [8].

^11^C- or ^18^F-choline (CH) positron emission tomography (PET)/CT seems an accurate tool for early PCa recurrence detection. CH is required for the biosynthesis of phosphatidylcholine, an essential cell membrane component. CH uptake increases in malignant tumors. There can, however, be overlap between radiolabeled CH uptake in prostatic tumors and benign prostatic tissue [9]. A meta-analysis reported pooled positive and negative predictive values of 70 and 85%, respectively, for ^11^C- and ^18^F-CH PET/CT performed at biochemical recurrence after RP [10]. CH PET/CT also appears to be highly accurate when performed in patients after EBRT, with 81% sensitivity and 93% specificity in a recent study [11, 12].

Gleason score and PSA kinetics, such as PSA doubling time (PSAdt) and velocity (PSAvel) are correlated to CH PET/CT detection rates [13]. After RP, thresholds for optimal PET/CT sensitivity are PSA level >1 ng/mL, PSAdt <6 months [14], and PSAvel >1 ng/mL/year [15]. There is no consensus for cutoff values after EBRT. Data interpretation is muddled by the fact that cytotoxic effects of EBRT occur over months or even years and do not affect all PSA-producing benign prostatic tissue. Also, the concomitant use of ADT can delay time to biochemical recurrence. PSA nadir is thus generally higher and occurs later after EBRT than after RP [16]. Recent studies have suggested that PSA kinetics have an impact on CH-PET/CT detection after EBRT, but that PSA levels at the time of PET/CT scan do not [17]. To the best of our knowledge, no studies have evaluated the impact of high nadirs on CH-PET/CT accuracy.

Thus, in order to challenge or confirm certain of these findings, we retrospectively analyzed a group of patients treated with EBRT to determine whether there were identifiable factors predictive of PET/CT positivity. We also evaluated the impact of taking PSA nadir into account when selecting patients for ^18^F-CH PET/CT after EBRT.

## Methods

### Patient population

**Fig. 1:**
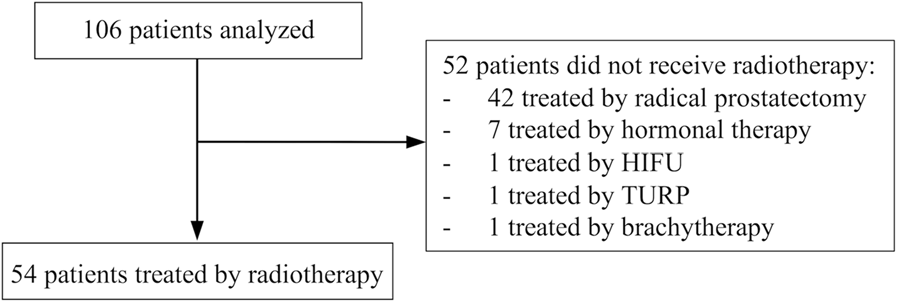
Flowchart of study population

In this retrospective study, 106 consecutive male patients diagnosed with PCa biochemical recurrence and referred to our center for restaging of disease with ^18^F-CH PET/CT between December 2010 and July 2015 were evaluated. Efforts were made to comply with the following PET/CT criteria: patients with PSAt <2 ng/mL, with Gleason score (GS) >7 and PSAdt <6 months, 2≤ PSAt ≤4 ng/mL and GS >7 and/or PSAdt <6 months, and PSAt >4 ng/mL (with any GS or PSAdt). Among these 106 patients, 65 (61%) were treated by radical prostatectomy (including 23 who further received salvage EBRT because of the absence of postoperative PSA decrease), 28 (26%) were treated by EBRT as initial treatment, 8 (8%) patients received ADT, 2 (2%) were treated with high-intensity focused ultra-sounds (HIFU), 2 (2%) were treated with transurethral resection of the prostate (TURP), and 1 (1%) was treated with brachytherapy. Three patients received salvage EBRT after ADT, HIFU, and TURP, respectively. Altogether, 54 (51%) patients were treated with EBRT (Fig. 1).

Data were collected from clinical and radiological files and recorded by the same investigator using a standardized form. Only patients with at least two PSA values since suspicion of relapse were included.

Study protocol was in accordance with the Declaration of Helsinki and local protocols.

### Imaging protocol

PET/CT was performed in non-fasting conditions [18]. An 8-min dynamic acquisition (8 × 1 min frames) centered on the pelvis was started immediately after intravenous injection of ^18^F-choline (3-3.5 MBq/kg). Following this, an acquisition was made from mid-thigh to skull base (five to six bed positions; 2 min 40s and 3 min 40s per bed position for normal weight (BMI <25) and for overweight patients (BMI ≥25), respectively). CT images were used for attenuation correction and topographic localization.

A lesion was considered abnormal when focal tracer accumulation was greater than background activity and consistent with prostate disease patterns.

### Studied parameters and definitions

Relative PSA (PSArel) was defined as the difference between PSA nadir and trigger PSA (PSAt), i.e., last PSA before PET/CT scan. PSA doubling time (PSAdt) was calculated by natural log of 2 (0.693) divided by the slope of the relationship between the log of PSA and time of PSA measurement for each patient [19]. If PSA levels decreased, PSAdt was assigned a value equal to 0. PSA velocity (PSAvel) was calculated with the following formula: (trigger PSA – PSA2)/Δ time, with PSA2 the PSA value at a Δ time from trigger PSA. PSAdt and PSAvel were calculated using the Memorial Sloan-Kettering Medical Center prostate cancer prediction tools [20]. Risk groups were determined according to the D’Amico classification [2].

Positive PET/CT results were considered true-positive when there was either confirmation of recurrence on histology of biopsies or surgical specimens, progressive disease (new uptake sites or increase in uptake at known sites) on follow-up PET/CT exams, repeated recurrence confirmation on conventional imaging (bone scan, MRI, CT scan), or biological and radiological response to local and/or systemic treatment with follow-up of more than 12 months.

### Statistical analysis

Quantitative variables were described with median and range and compared between PET/CT positive and negative groups by Mann-Whitney test, whereas qualitative variables were described with numbers and percentages and compared between PET/CT positive and negative groups by chi-square test (Fisher exact test if needed).

Comparisons between trigger and relative PSA values were done using Wilcoxon signed rank test for paired samples in both initial and salvage EBRT groups. Comparisons of PSA values between initial and salvage EBRT groups were done using Mann-Whitney tests. Two-by-two comparisons of PSAt according to the sites of relapse were done using Mann-Whitney tests.

Concerning PET/CT positivity, univariate tests were first performed to detect possible predictive factors. To detect the best predictor of PET/CT positivity between PSAt and PSArel, Akaike information criterion (AIC) was used.

For multivariate analysis, two logistic regressions were computed with PSA values, one with PSAt and one with PSArel because of their collinearity, as well as with PSA doubling time, PSA velocity, and D’Amico risk group. The best two models were chosen with stepwise algorithm, using the AIC criteria.

Moreover, ROC curves of PSAt and PSArel were estimated for their prediction of PET/CT results using the AUC value. Youden’s index was used to determine the best cutoff in terms of both sensitivity and specificity.

*P* < 0.05 was considered significant. All analyses were performed with R, version 3.1.2 (R Foundation for Statistical Computing: https://www.r-project.org/) and Graphpad software.

## Results

### Patient characteristics

Age, clinical TNM stages, Gleason scores, D’Amico risk groups, and initial PSA levels of the 54 patients treated by EBRT are detailed in Table 1. At the time of PET/CT scanning, no patients had documented metastatic disease and 12 (22%) were receiving ADT. Three patients were receiving “adjuvant” hormonal therapy combined with and then pursued for 2 to 3 years after radiotherapy (because of an initial high risk of recurrence). The other nine patients were under hormonal therapy for rising PSAs with undocumented metastatic disease (despite repeated conventional imaging) and could be described as micro-metastatic.

**Table 1:** Characteristics of patients with and without positive ^18^F-choline PET/CT

	Total	PET-positive	PET-negative	*p* value
*N*	54	40	14	
Age at diagnosis (year)	62 (51–78)	61.5 (51–77)	62 (51–78)	0.57
Cancer characteristics				
Initial PSA (ng/mL)	9.84 (4–180)	9.58 (4–180)	10.51 (4.26–180)	0.86
Initial Gleason score	7 (5–9)	7 (5–9)	7 (6–9)	0.40
Gleason score ≥7	36 (67)			
TNM				0.79
T11	11 (20.5)	8 (20)	4 (29)	
T2	18 (33)	13 (32.5)	5 (35.5)	
T3	24 (44.5)	19 (47.5)	5 (35.5)	
Other T (is, x)	1 (2)			
N0	34 (63)	24 (60)	10 (71)	
N1	2 (4)	2 (5)	0	
N2	1 (2)	1 (2.5)	0	
Nx	17 (31)	13 (32.5)	4 (29)	
D’Amico risk group				*0.004*
High	38 (70.5)	31 (77.5)	7 (50)	
Intermediate	11 (20.5)	4 (10)	7 (50)	
Low	5 (9)	5 (12.5)	0	
Initial treatment				0.12
RP	23 (43)	16 (40)	7 (50)	
EBRT	28 (52)	23 (57.5)	5 (36)	
Other^a^	3 (5)	1 (2.5)	2 (14)	
PSA values				
PSA nadir (ng/mL)	0.31 (0.01–13.31)	0.30 (0.01–11.26)	0.50 (0.01–13.31)	0.40
Pre-PET/CT PSA (trigger PSA)	7.85 (0.47–111.60)	9.14 (2.04–111.60)	4.25 (0.47–83.40)	*0.015*
Relative PSA	6.05 (0.24–104.59)	8.06 (1.85–104.59)	2.34 (0.24–82.97)	*0.0005*
PSA kinetics				
PSAdt in the last 12 m before PET/CT (months)	5.4 (0.6–91.6)	4.7 (1.7–27.7)	7.7 (0.6–91.6)	0.15
PSA velocity (ng/mL/year)	6.40 (0.50–104.40)	8.10 (0.60–104.40)	3.05 (0.50–86.10)	*0.01*
PET/CT				
ADT at time of PET/CT	12 (22)	11 (27.5)	1 (7)	0.11
Time from initial treatment to PET/CT (months)	83 (4–222)	86 (4–222)	77 (11–123)	0.27

All medians are followed by (min–max) interval

*ADT* androgen deprivation therapy, *EBRT* external beam radiation therapy, *PSA* prostate-specific antigen, *PSAdt* PSA doubling time, *RP* radical prostatectomy

^a^Other: hormonal therapy (*n* = 1), high-intensity focused ultrasounds (*n* = 1), transurethral resection of the prostate (*n* = 1)

### PSA parameters

**Fig. 2:**
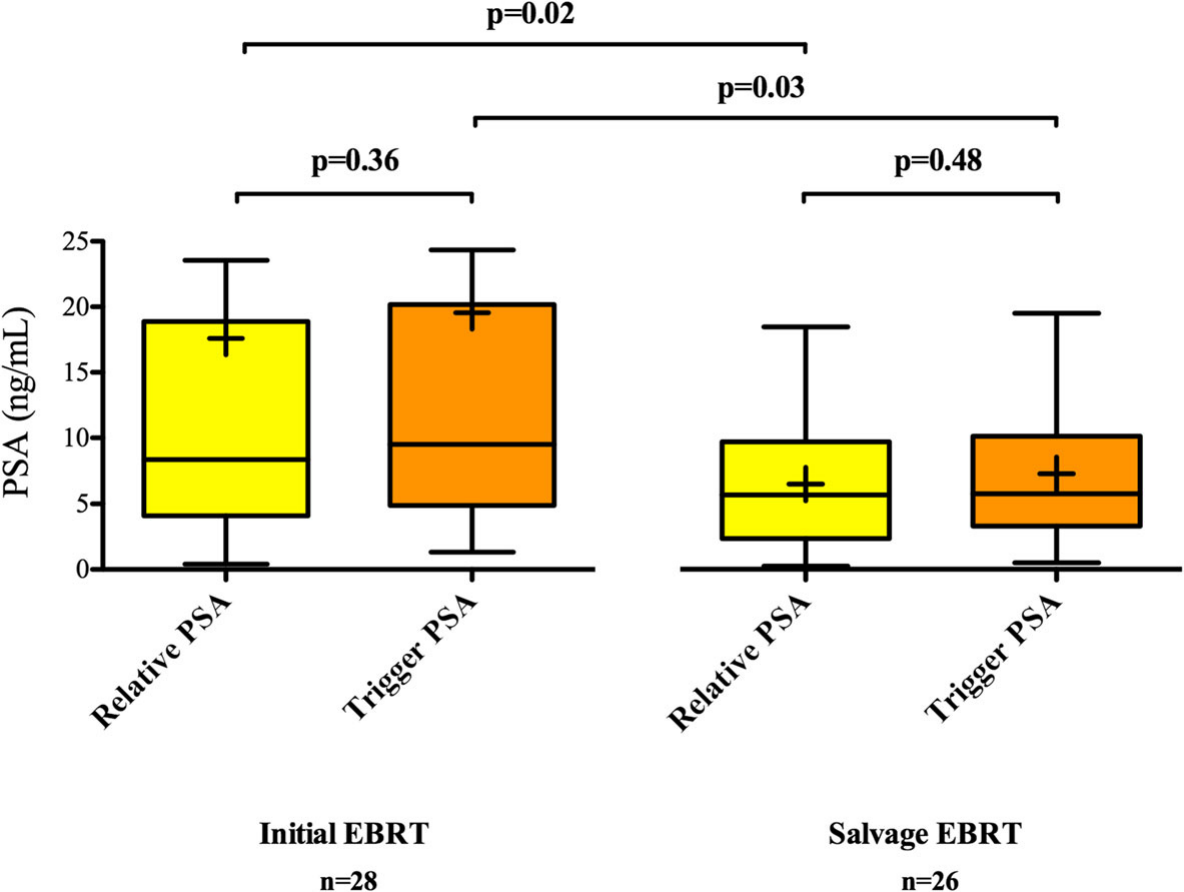
Relative and trigger PSA values of patients treated with external beam radiation therapy (EBRT). *Boxplots* represent median and interquartile ranges; *crosses* represent means

Median PSA nadir was 0.31 (0.01–13.31) ng/mL, equivalent to a median 4.2 (0.1–88.7) % of PSAt. Median relative PSA was 6.05 (0.24–104.59) ng/mL. Among the 54 patients, no significant differences between PSArel and PSAt values were found (Fig. 2). Sixteen patients had PSA nadir >1 ng/mL and their median time to biochemical recurrence was significantly shorter than patients with nadir ≤1 ng/mL (7 (2–58) vs. 39 (3–228) months; *p* = 0.001).

### ^18^F-choline PET/CT results

^18^F-CH PET/CT detected PCa recurrence in 40 (74%) patients. Recurrence was local and/or regional in 29 patients (including 12 with prostatic fossa involvement, 14 with pelvic nodal involvement, and three with both) and distant in 15 patients. Among these 15 patients, 10 presented only bone metastases, one presented both bone and soft tissue metastases, and four patients presented both regional nodal and distant recurrence (i.e., distant lymph node involvement, bone and/or visceral lesions). There were no significant differences between median trigger or relative PSA values of patients with local or distant recurrences. Distant recurrences were found in patients with PSAt both superior and inferior to 4 ng/mL. No false positive scans were observed.

### Recurrence confirmation and subsequent treatment

Six (15%) positive PET/CT scans were confirmed by lymph node or metastasis pathology, three (7.5%) by TRUS-guided biopsy, 19 (47.5%) by response to treatment, and 12 (30%) by conventional imaging or repeated PET/CT exams, with a median follow-up time after ^18^F-CH PET/CT of 26 (5–61) months for all patients.

Among the 19 patients with recurrence confirmed by response to treatment, 15 were treated with hormonal therapy (HT), two with radiotherapy (EBRT), one with both HT and EBRT, and one with chemotherapy.

Out of the 40 patients with positive PET/CT scans, 27 (67.5%) were treated with ADT (including one patient also treated with nodal EBRT), five (12.5%) were treated with radiotherapy alone (EBRT or cyberknife), three (7.5%) with chemotherapy, one (2.5%) with orthopedic surgery, and four (10%) were followed up and treated only at further progression.

### Impact of trigger PSA, PSA kinetics, and PSA nadir on PET/CT detection rates

Differences in PSAt and PSArel between patients with positive or negative ^18^F-CH PET/CT are shown in Table 1. In univariate analysis, patients with positive PET/CT had significantly higher PSAt (*p* = 0.015), PSArel (*p* = 0.0005) levels and higher PSAvel (*p* = 0.01) than patients with negative PET/CT scans. There were no significant differences in terms of PSAdt or PSA nadir between both groups. AIC model comparison found that PSArel was a better predictor of positive PET/CT than PSAt (AIC: 46.4 vs 55.7, respectively).

For multivariate analysis, whatever the PSA (trigger or relative) entered into the model, the final selected model was the one with only PSA velocity (HR (+1 ng/mL/year) 1.03 CI95% 0.99–1.07, *p* value = 0.12) and D’Amico risk group (high vs. intermediate/low: HR 4.36 CI95% 1.12–17.00, *p* value = 0.032).

**Fig. 3:**
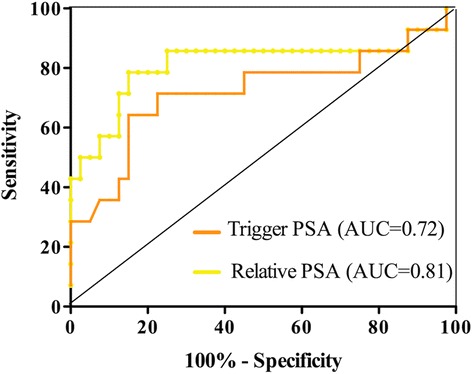
ROC analysis of the optimal cutoff of trigger and relative PSA values for highest PET/CT accuracy

ROC analysis for prediction of ^18^F-CH PET/CT positive scans found the best cutoff point for PSArel to be 4.09 ng/mL (sensitivity = 85%, specificity = 78%), and AUC was 0.81 (Fig. 3).

The percentages of positive ^18^F-CH PET/CT scans were 0% (0/4 patients), 71% (5/7 patients), and 81% (35/43 patients) for PSAt <2 ng/mL, 2≤ PSAt ≤4 ng/mL, and PSAt >4 ng/mL, respectively. Seven (13%) patients changed subgroups when PSArel was taken into account instead of PSAt: 5/14 (36%) patients with negative PET/CT were down-graded from their PSA subgroup (2 from the “>4” to the “< 2” subgroup and 3 from the “>4” to the “2 ≤ PSA ≤ 4” subgroup) and 2/40 (5%) patients with positive PET/CT were down-graded. Detection rates were 14% (1/7 patients) for PSArel <2 ng/mL, 50% (5/10 patients) for 2≤ PSArel ≤4 ng/mL, and 92% (34/37 patients) for PSArel >4 ng/mL.

**Fig. 4:**
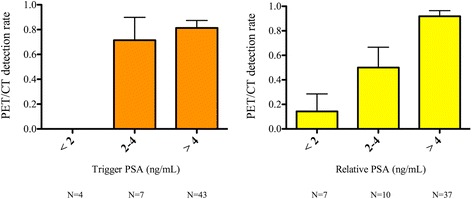
^18^F-CH-PET/CT positivity according to trigger and relative PSA subgroups. *Boxes* represent PET/CT detection rates; *upper bars* represent standard deviation

As shown in Fig. 4, when PSArel is superior to 4 ng/mL, relative PSA allows for better discrimination between positive and negative PET/CT exams as 92% of patients with PSArel >4 ng/mL had a positive exam. Conversely, when PSAt is inferior to 2 ng/mL, PSAt discriminates between positive and negative exams better than PSArel as all patients with PSAt <2 ng/mL had a negative exam. Patients in the 2–4 PSA subgroup, have a lower probability of positive exams (50 vs. 71%) when taking PSArel into account rather than PSAt. It is likely that other predictive factors are necessary to consider scanning these patients.

### Subgroup analysis of patients treated with initial EBRT and salvage EBRT

Among patients treated with initial EBRT and those treated with salvage EBRT, PSArel and PSAt values were not significantly different (Fig. 2). However, median PSAt and PSArel values were both significantly higher in patients initially treated with EBRT than those treated with salvage EBRT (9.39 (1.32–111.60) vs. 5.53 (0.47–18.62) ng/mL, *p* = 0.03 and 8.49 (0.41–104.59) vs. 5.26 (0.24–17.62) ng/mL, *p* = 0.02, respectively).

There was no significant difference in median PSA nadir between the salvage EBRT group and the initial EBRT group (median of 0.22 and 0.48 ng/mL, respectively, *p* = 0.32).

When comparing patients with positive and negative PET/CT scans, the significant differences of PSAt and PSArel found among the 54 patients were only found in the salvage EBRT group (Table 2).

**Table 2:** PSA parameters and Gleason scores of patients with negative or positive PET/CT, in subgroups of patients treated with initial or salvage radiation therapy

	Total	Positive PET/CT	Negative PET/CT	*p* value
Initial radiotherapy	*N* = 28	*N* = 23	*N* = 5	
Trigger PSA	9.39 (1.32–111.60)	9.50 (2.04–111.60)	8.50 (1.32–83.40)	0.76
Relative PSA	8.49 (0.41–104.59)	8.59 (2.03–104.59)	4.00 (0.41–82.97)	0.47
Gleason score ≤6	12 (43)	10 (44)	2 (40)	1.00*
Gleason score >6	16 (57)	13 (56)	3 (60)	
PSAdt >6 months	9 (32)	7 (30)	2 (60)	1.00*
PSAdt ≤6 months	19 (68)	16 (70)	3 (40)	
Salvage radiotherapy	*N* = 26	*N* = 17	*N* = 9	
Trigger PSA	5.53 (0.47–18.62)	7.81 (2.60–18.62)	3.85 (0.47–15.01)	0.009
Relative PSA	5.26 (0.24–17.62)	7.51 (1.85–17.62)	1.69 (0.24–5.09)	0.0004
Gleason score ≤6	7 (27)	5 (29)	2 (22)	1.00*
Gleason score >6	19 (73)	12 (71)	7 (78)	
PSAdt >6 months	15 (58)	8 (47)	7 (80)	0.22*
PSAdt ≤6 months	11 (42)	9 (53)	2 (20)	

*PSAdt* PSA doubling time

*Fisher’s exact test; all others: Mann-Whitney test

## Discussion

Our study suggests that ^18^F-CH PET/CT detection rates are positively related to relative PSA levels as well as trigger PSA levels in patients treated with EBRT, especially in patients treated with EBRT as salvage therapy after RP.

For PSA values above 4 ng/mL, taking relative PSA into account instead of trigger PSA increased PET/CT positivity. However, for PSA values below 2 ng/mL, trigger PSA allowed for better discrimination of negative PET/CT exams compared to relative PSA with detection rates dropping from 14 to 0%, respectively. On the basis of these results, the usefulness of taking the nadir into account for PET/CT indication seems limited.

Indeed, early and reliable detection of relapse can guide therapy and justify local treatment such as radiotherapy or surgery, and delay hormonal therapy. Thus, PET/CT should not, theoretically, primarily benefit patients with high PSA values, but rather those with low PSAs [21]. However, for very low PSAs (<1 ng/mL), studies have so far reported variable detection rates from 0 to 49%, usually in heterogeneous groups of patients treated with either RP or EBRT [11, 22–24]. Our detection rates for low PSA values (despite Gleason score and PSAdt selection criteria) were weak, but further analysis is limited by the small number of patients.

Similarly to our study, some authors found that PSA levels were not predictive of the site of recurrence with distant and local recurrences in all subgroups of PSA [25, 26]. This underlines the importance of PET/CT scans for cases in which local treatment is discussed to determine sites of recurrence and adapt treatment as biological analyses alone are insufficient.

Many authors have studied the impact of trigger PSA and PSA kinetics on ^11^C- and ^18^F-CH PET/CT positivity, but generally studies have grouped patients treated with EBRT with those treated with RP. Among those distinguishing EBRT from RP patients, Bertagna and al. studied 70 patients treated with EBRT and suggested an optimal cutoff of 2 ng/mL of trigger PSA with best PET/CT sensitivity of 81.8% and specificity of 92.9%. As opposed to patients treated with RP, they did not find a statistical correlation between PSA values and PET/CT results in the subgroup treated with EBRT [12]. Chondrogiannis et al. only found an impact of trigger PSA on PET/CT detection rates in a study of 34 patients initially treated with EBRT and an overall detection rate of 80%, similar to ours [25]. Later work by Ceci et al. found an impact of PSA kinetics (PSAdt and PSAvel) on PET/CT detection rates in 140 patients with recurrence after EBRT, but no impact of trigger PSA (*p* = 0.20) [17].

We found impacts of trigger PSA and PSAvel on PET/CT positivity, but not of PSAdt. Likewise, Gleason score did not seem to influence our PET/CT results, which diverges from the results of a recent study evaluating Gleason score impacts on PET/CT detection rates in patients treated with EBRT, RP, or ADT [27]. As shown by prior studies [22, 24, 25], ADT at the time of CH-PET/CT did not have a significant effect on detection rates in our series.

To our knowledge, no studies have taken into account patients’ PSA nadir after EBRT. No target post-EBRT PSA nadir is established, but, as we found in our study, it is suggested that PSA nadir ≤1 ng/mL is correlated to longer disease-free survival [28]. Intuitively, PSA nadir should have a bigger impact on PET/CT results in post-EBRT patients with low PSAs as the subtraction of the nadir from a low trigger PSA would diminish PSA elevation and thus the probability of positive PET/CT scan. We could not determine this in multivariate analysis of our series because of the limited number of subjects with low PSA.

Our study is limited by its retrospective design, precluding complete data collection, and its small sample size. As others have mentioned, validation of PET/CT results is problematic as the gold standard, histological analysis of each detected lesion, is neither ethical nor practical, and is usually performed only in patients with positive PET/CT [29]. Nevertheless, the prolonged follow-up time of our cohort, with both clinical and radiological examinations (repeated PET/CT and other types of imagery), reduced the likelihood of false negatives and positives. Patients treated with salvage EBRT were the only sub-group of patients with an impact of PSAt and PSArel values on PET/CT accuracy, but this group also had significantly lower PSAt and PSArel values than those treated with initial EBRT, which could induce bias.

Finally, our recommendations for PET/CT intended to select for patients with a high a priori probability of PET/CT positivity, which may be regarded as a bias. A few patients in our cohort had very high trigger PSAs and median trigger PSA was slightly higher than those found in the post-EBRT studies described previously [17, 25]; however, none of our patients had documented metastatic disease but those with very high PSA values could probably be considered micro-metastatic. Our overall detection rate of 74% was also higher than the 62% in a recent meta-analysis by Fanti et al. [29] but similar to other studies of post-EBRT patients [25].

Recent studies suggest that PMSA, a newer tracer, is more accurate than choline for the diagnosis of prostate cancer recurrence, especially for low PSA values, but the use of this tracer is still restricted to large academic centers [30–34].

Further prospective studies are necessary to determine the impact of PSA nadir on ^18^F-CH or other radiotracer PET/CT detection rates.

## Conclusions

In this routine clinical setting study of patients with rising PSA values after curative EBRT, ^18^F-CH PET/CT detection rates were high and were correlated to trigger and relative PSA, the latter being the best predictor of a positive exam. For high PSAs (>4 ng/mL), relative PSA discriminated between positive and negative PET/CT scans more optimally than trigger PSA. For low PSAs (<2 ng/mL), trigger PSA was most relevant and it does not seem necessary to consider PSA nadir in these patients. Larger series with intermediate PSA values need to be studied to fully apprehend nadir impact.

## Abbreviations

^18^F-CH: ^18^F-fluorocholine
ADT: Androgen deprivation therapy
AIC: Akaike information criterion
AUC: Area under the curve
BMI: Body mass index
EBRT: External beam radiation therapy
GS: Gleason score
HIFU: High-intensity focused ultrasounds
PCa: Prostate cancer
PET/CT: Positron emission tomography/computed tomography
PSA: Prostate-specific antigen
PSAdt: PSA doubling time
PSArel: Relative PSA
PSAt: Trigger PSA
PSAvel: PSA velocity
ROC: Teceiver-operating characteristic
RP: Radical prostatectomy
TRUS: Rrans-rectal ultrasound
TURP: Transurethral resection of the prostate
